# Additively manufactured patient-specific prosthesis for tumor reconstruction: Design, process, and properties

**DOI:** 10.1371/journal.pone.0253786

**Published:** 2021-07-14

**Authors:** Maryam Tilton, Gregory S. Lewis, Michael W. Hast, Edward Fox, Guha Manogharan

**Affiliations:** 1 Department of Mechanical Engineering, Pennsylvania State University, University Park, Pennsylvania, United States of America; 2 Department of Orthopaedics and Rehabilitation, Pennsylvania State University, Hershey, Pennsylvania, United States of America; 3 Biedermann Lab for Orthopaedic Research, Department of Orthopaedic Surgery, University of Pennsylvania, Philadelphia, Pennsylvania, United States of America; University of Zaragoza, SPAIN

## Abstract

Design and processing capabilities of additive manufacturing (AM) to fabricate complex geometries continues to drive the adoption of AM for biomedical applications. In this study, a validated design methodology is presented to evaluate AM as an effective fabrication technique for reconstruction of large bone defects after tumor resection in pediatric oncology patients. Implanting off-the-shelf components in pediatric patients is especially challenging because most standard components are sized and shaped for more common adult cases. While currently reported efforts on AM implants are focused on maxillofacial, hip and knee reconstructions, there have been no reported studies on reconstruction of proximal humerus tumors. A case study of a 9-year-old diagnosed with proximal humerus osteosarcoma was used to develop a patient-specific AM prosthesis for the humerus following tumor resection. Commonly used body-centered cubic (BCC) structures were incorporated at the surgical neck and distal interface in order to increase the effective surface area, promote osseointegration, and reduce the implant weight. A patient-specific prosthesis was fabricated using electron beam melting method from biocompatible Ti-6Al-4V. Both computational and biomechanical tests were performed on the prosthesis to evaluate its biomechanical behavior under varying loading conditions. Morphological analysis of the construct using micro-computed tomography was used to compare the as-designed and as-built prosthesis. It was found that the patient-specific prosthesis could withstand physiologically-relevant loading conditions with minimal permanent deformation (82 *μm* after 10^5^ cycles) at the medial aspect of the porous surgical neck. These outcomes support potential translation of the patient-specific AM prostheses to reconstruct large bone defects following tumor resection.

## 1. Introduction

In recent years, the inherent layer-by-layer characteristic of additive manufacturing (AM), commonly referred to as 3D printing, has provided new opportunities to meet medical needs [1]. One of the main areas of interest in medical applications of AM is the reconstruction of large bone defects. In general, large bone defects are caused by disease, trauma, or tumor resection. These large defects cannot be healed through natural bone regeneration, and require surgical intervention and regenerative therapies to assist with the healing process [2]. Reconstruction of large bone defects remains a major challenge due to ineffectiveness of commercially available “standard” implants to accommodate high patient-specific variability in anatomy, bone quality, daily activities and biologic healing capacity. This challenge is evident from the relatively high rate of complications associated with reconstruction of large bone defects [3–5].

Osteosarcoma is an invasive bone tumor which primary occurs during the pubertal growth spurt followed by late adulthood (age of 70–85 years) [6, 7]. The proximal humerus is one of the most common sites for osseous sarcoma and osteosarcoma [8]. Current reconstruction approaches after tumor resection include a cemented allograft-prosthesis composite, osteoarticular allograft, and modular tumor prosthesis. In recent decades, osteoarticular allografts are uncommon due to the advent of improved modular prostheses [9]. In addition, higher rate of fractures and lengthy time for alignment during operation are prevalent in osteoarticular allografts. While there are several reconstruction approaches there is no consensus on the ideal treatment approach [8]. Although endoprostheses have been reported to have relatively lower complication rates [10], reconstruction of the humerus after tumor resection continues to be a challenge because of several common complications including proximal displacement, subluxation of the proximal head, and aseptic loosening of the stem [8, 11].

Pediatric patients diagnosed with osteosarcoma face a lower survival rate and higher rate of revision surgery when compared to adult patients [6]. A critical design-based challenge in addressing osteosarcoma in pediatric surgery is the reconstruction of limb after tumor resection with off-the-shelf implants that are primarily designed based on adult anthropometric measures. Often those implants result in more pronounced leg or arm–length discrepancy after endoprosthesis. In some cases, surgeons consider amputation as the safest and most suitable option for these patients when a properly sized and shaped implant does not exist.

On the other hand, unique design and manufacturing capability of AM offers the potential for a systematic design, fabrication and evaluation of new workflow to produce patient-specific implants for tumor reconstruction. Patient-specific implants could potentially reduce the risk of intra- and post- operative complications. It has been proposed that tailoring surgical options to the patient’s anatomic defects and functional requirement could substantially reduce or eliminate intra- and post- operative complications [9]. Previously published reports on AM orthopaedic implants are mainly focused on maxillofacial [12], clavicle [13], hip [14, 15] and knee [16] joint replacements.

**Fig 1:**
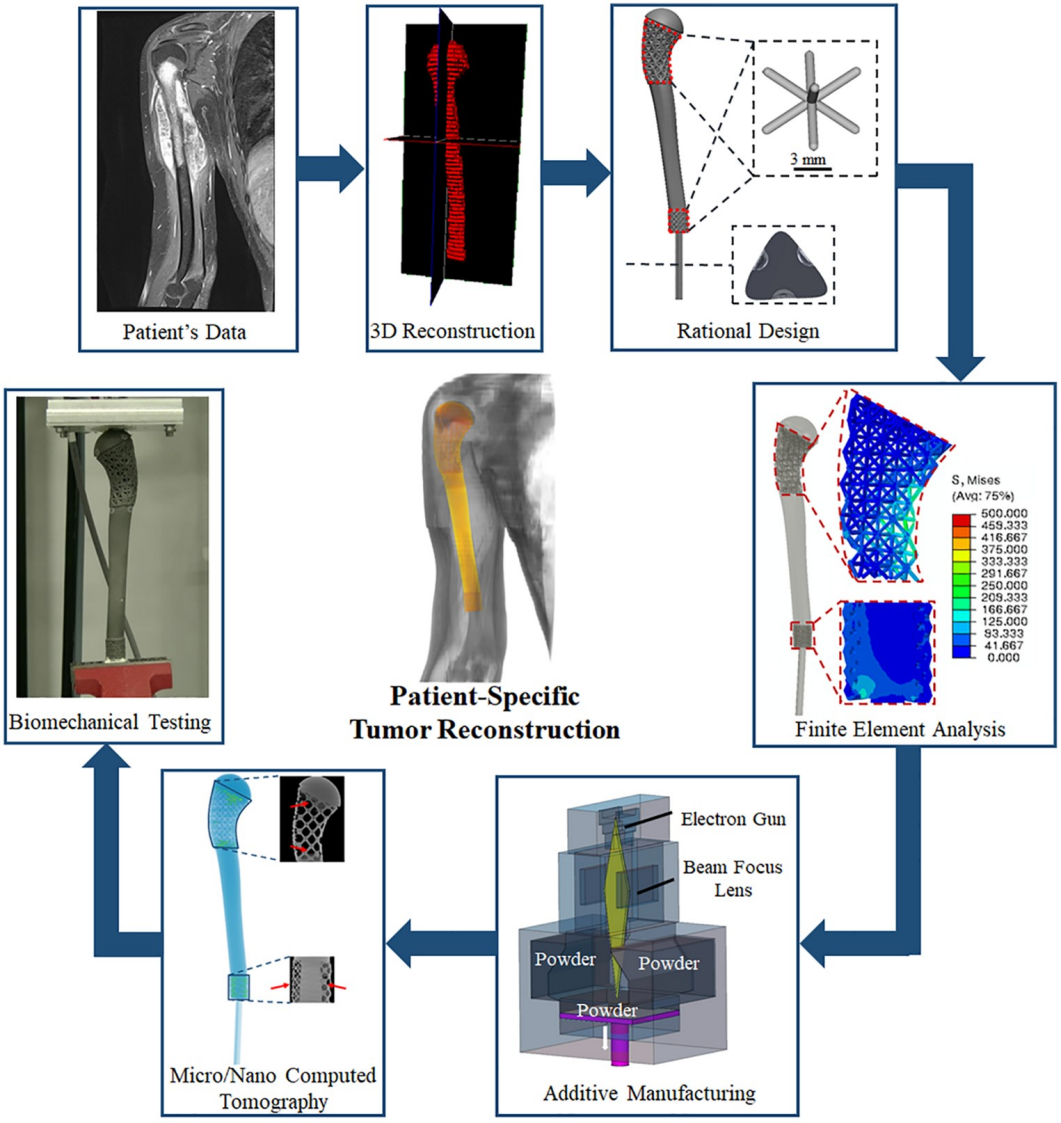
Design workflow for patient-specific AM prosthesis for reconstruction of large bone defects after tumor resection including computed tomography (CT) of metal AM prosthesis.

To the best of the authors’ knowledge, this is the first reported study on the reconstruction of proximal humerus after tumor resection using AM (Fig 1). The aim of this study was to develop a systematic procedure for the design-biomechanical FEA analysis-AM of patient-specific prosthesis which can achieve required biomechanical strength and anatomical fit for the proximal humerus after tumor resection.

## 2. Methods

### 2.1. From patient’s data to implant design

**Fig 2:**
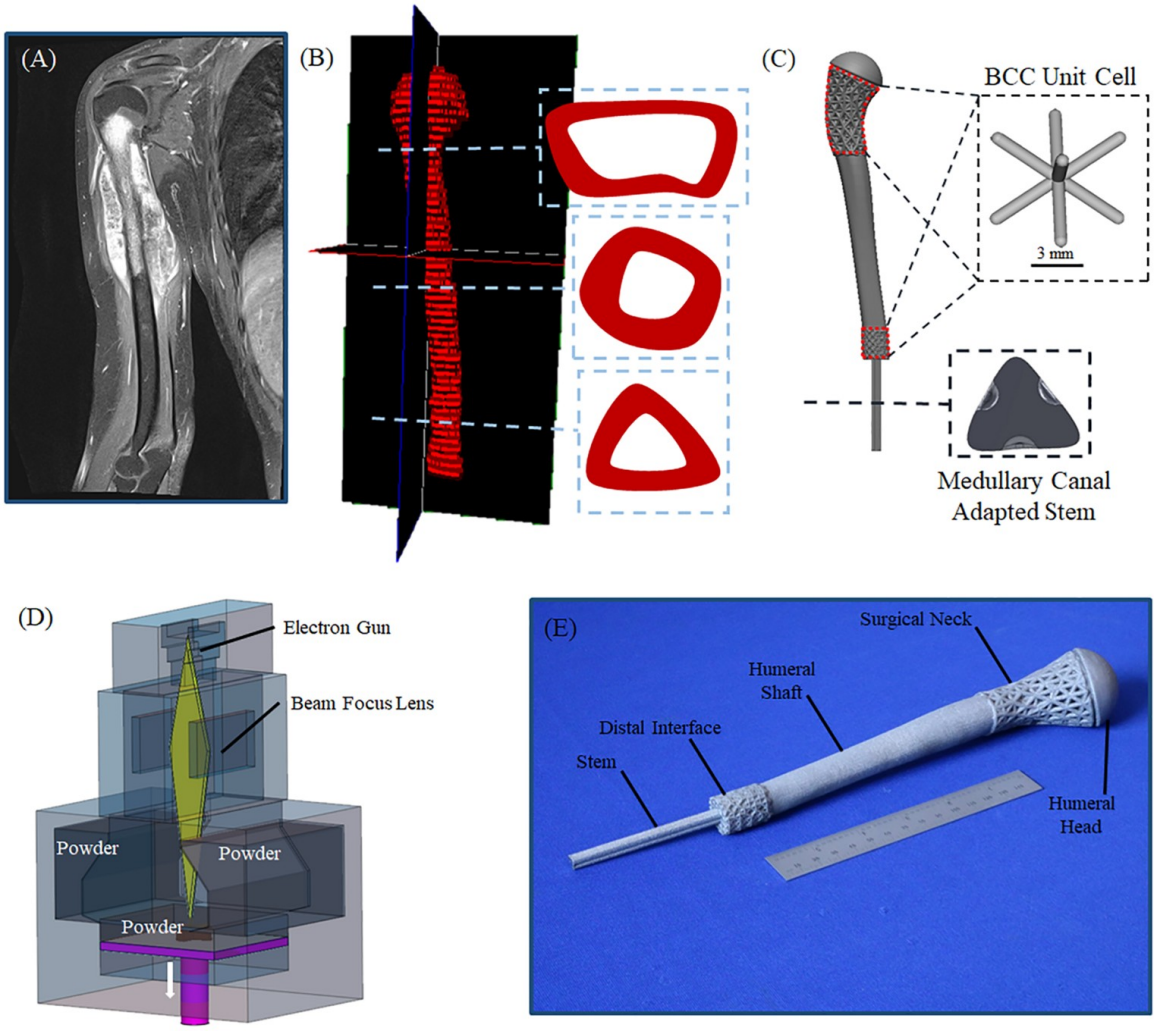
Patient’s data to design workflow; (A) patient’s MRI DICOM data, (B) reconstructed 3D anatomy of the proximal humerus with graded cross-section from distal to proximal aspect, and (C) designed patient-specific prosthesis. (D) Schematic diagram of the EBM process, and (E) final patient-specific AM prosthesis for tumor reconstruction.

This study was approved by CATS IRB—Penn State’s Centralized Application Tracking System for Institutional Review Board applications. This study included the use of pediatric patient image data retrospectively. Additionally, in the associated approved IRB protocol (IRB study #: 005099), waiver of informed consent was requested. IRB-approved magnetic resonance imaging (MRI) data of a 9-year-old patient diagnosed with proximal humerus osteosarcoma was obtained (Fig 2A). In the present study, the approved IRB protocol included waiver of informed consent and existing images were used retrospectively. In this study, standard clinical MRI protocol was followed using 1.5-Tesla MRI scanner (Siemens, USA) with slice thickness of 7.2 mm. Consequently, preoperative DICOM data from the ipsilateral aspect of the patient was used for image segmentation and 3D anatomy reconstruction in Avizo software (version 9.0, Amira-Avizo, FEI, Thermo Fisher Scientific, USA). DICOM image stack with original image quality of 32 bit was imported into Avizo. The pixel size for every frame (or image) was 0.3125 to 0.3125. 3D anatomy reconstruction included use of frame-by-frame thresholding method for segmenting the ipsilateral humerus in order to create polygonal surface model. Additional surface smoothing and triangular reduction was performed on the 3D polygonal surface model. Later, the reconstructed 3D model of the humerus was imported into Geomagic Design X (3D Systems) to develop parametric design on a polygonal surface model of the bone.

The geometrical variations in cross-sections were incorporated into the design of the prosthesis shaft (Fig 2B). The distal cut-off plane was chosen to be 30 mm inferior to the last frame (i.e., image) malignant bone was observed. The radius of the humeral head (17.6 mm) was measured from the reconstructed 3D anatomy for the design of the proximal head in the AM prosthesis. In order to improve the bone-implant interface and facilitate for bone ingrowth, porous structures were introduced at the shell of the distal interface of the AM prosthesis (Fig 2C). ElementPro (nTopology, Inc. USA) was used to create BCC (body-centered cubic) [17] porous structures with unit cell dimensions of 7 mm and 3 mm at the surgical neck and distal-cortical contact, respectively. The reason behind this selection was that BCC topology has previously been extensively studied for similar applications [17–19]. BCC-based structures exhibit high elastic modulus and yield strength with small porosity which make BCC a good candidate for reconstruction of bone [17]. Additionally, unlike other beam-based topologies (e.g., Tetrahedron and octet truss), BCC topology offers large pore size for similar mechanical properties [17]. A uniform strut thickness of 1 mm was employed at the distal interface. A vector-based gradient was employed for the strut thickness (0.8–2 mm) at the surgical neck. Nominal porosity of 76.8% and 69.8% were obtained at the surgical neck and distal interface, respectively, based on the Eq 1, where *V*_*p*_ corresponds to the volume of the porous media and *V*_*s*_ is the volume of the replacing solid media. The target porosities of cellular regions in the designed implant were chosen to mimic those of trabecular bone (50–95%) [20–22] and to facilitate working space for surrounding soft tissue attachments. Large pore size of the BCC structure at the surgical neck of the designed prosthesis was aimed to facilitate suturing of tendons into the cellular channels and bone grafting as needed.


$$P\%=(1-\frac{V_{p}}{V_{s}})\times 100$$ (1)

### 2.2. Manufacturing

The patient-specific prosthesis was fabricated using an electron beam melting (EBM) machine (ArcamEBM, GE Additive Company, USA) which is a powder bed fusion technique (Fig 2D). Stereolithographic (STL) file of the designed prosthesis was imported into Magics software 21.0 (Materialise, Leuven, Belgium) for rendering and support structure generation. Commercially available biocompatible Ti-6Al-4V (Grade 5) spherical powder (D10 = 52, D50 = 71, D90 = 102, $\text{apparent}\;\text{density}=2.47\;\frac{g}{cm^{3}}$, and oxygen content of 0.13%) with size distribution of 45–106 μm was used as the feedstock material. The powder was deposited in layers of 90 μm thickness. The beam overlap was 0.2 mm with effective contour, support, and melt speed of 17.18, 50, and 500 mm/s, respectively. Total height of the build was 325.08 mm (Fig 2E). Sandblasting was performed on the porous areas of the prosthesis in order to attempt to remove the trapped powders inside the pores.

### 2.3. Cyclic loading

**Fig 3:**
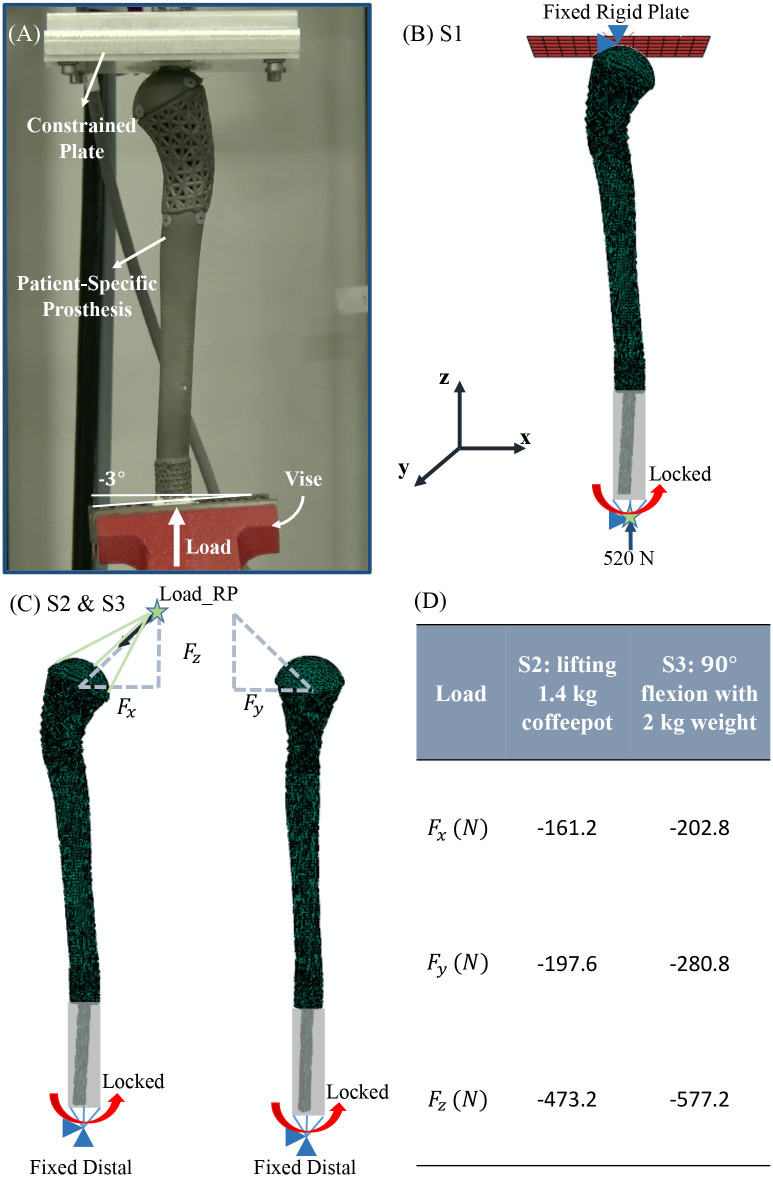
(A) Biomechanical testing (cyclic loading) of AM prosthesis for tumor reconstruction. (B-D) Applied boundary conditions in the FEA models; the grey region indicates where the stem (green) was fixed to a reference point at the bottom of the images; (B) S1 which mimics the previously established biomechanical test setup, and (C-D) S2 and S3 boundary conditions with applied multi—directional joint reaction forces [33] based on the previous in-vivo study.

The fabricated AM prosthesis was tested under cyclic loading in a universal test frame (ElectroForce 3550, TA Instruments). A custom-made fixture was prepared to emulate physiologically relevant loading conditions by securing the prosthesis stem in the test frame (Fig 3A). Prior to the experiment, the mechanical test setup was calibrated using a synthetic humerus bone. Surgical cement was employed to fill the interface between the implant’s stem and custom fixture. The test frame was equipped with a vertically actuated load cell (±15 kN). Petroleum jelly was applied to the plate at the humeral head contact to minimize the effects of contact friction forces. The prosthesis was cyclically loaded at a frequency of 4 Hz with a peak load of 520 N for 10^5^ cycles [23]. Here, 520 N applied load corresponds to the patient’s body weight (BW). Biomechanical evaluation of total shoulder arthroplasty (TSA) implants is often conducted assuming typical BW [24]; therefore, withstanding full body weight was considered sufficient for the designed prosthesis. And, the 10^5^ cycles correspond to 8–10 weeks of rehabilitation [25, 26]. Stiffness of the construct was calculated based on the slope of the linear region of force-displacement plots at increments of 100 cycles of the load-displacement curve.

### 2.4. Finite element modeling & analysis

FEA of the prosthesis under quasi-static loading was performed in Abaqus/CAE v6.13 (Simulia, Dassault Systems, Providence, USA). Due to limitations in extracting parametric CAD models of highly complex BCC porous structures for computationally efficient finite element modeling (FEM), polygonal surface model (i.e., STL) was extracted from ElementPro. An open source program (GMSH) [27] with Delaunay algorithm was used for FE mesh generation. A mesh convergence study was performed by varying the maximum element edge length from 0.5 to 4 mm. As a result, the FE model was meshed with 422,227 linear tetrahedron elements (C3D4) which was within 4% convergence. Subsequently, meshed model was imported into Abaqus as an orphan-mesh for further FE post-modeling. Linear elastic isotropic material properties of biocompatible Ti-6Al-4V (ELI), E = 120 GPa and ν = 0.3, were assigned to the meshed elements.

Over the course of five years during clinical follow-up, patient’s body weight was within the range of 49–54 kg. Hence, 520 N body weight was used to determine loads used in FEAs. In order to predict the performance of the designed prosthesis, three different loading and boundary conditions, based on patient’s body weight, were investigated via FEA (Fig 3B–3D) to simulate: (S1) previously established in-vitro biomechanical testing setup [28–31] (Fig 3A and 3B), (S2) patient lifting a 1.4kg coffeepot during early rehabilitation (Fig 3C and 3D), and (S3) flexion at 90° with 2kg weight (Fig 3C and 3D).

In the case of S1 which mimics the biomechanical test setup, stem of the prosthesis was kinematically coupled to a Reference Point (Load-RP) that was constrained in five degrees of freedom and only free for translational motion along the z-axis. The rigid body plate (mimicking the machine platen) that contacts the proximal head of the prosthesis was constrained in all six degrees of freedom. Interaction between the head of the prosthesis and the rigid plate was modeled as isotropic coulomb friction with a coefficient of 0.1 [32].

In S2 and S3 FEA, stem of the prosthesis was kinematically coupled to a reference point which was constrained in all degrees of freedom. In these models, Load-RP was located proximally and kinematically coupled to the surface nodes of the proximal head. In all the FE models, boundary conditions similar to the experiment was implemented; this included constraining the distal about rotation. Joint reaction forces reported by previous in-vivo study [33] for common shoulder activities (S2 and S3) were applied at the Load-RP points. All the FEA was performed via standard implicit static solver in Abaqus.

### 2.5. Characterization

#### 2.5.1. X-ray micro-computed tomography

**Fig 4:**
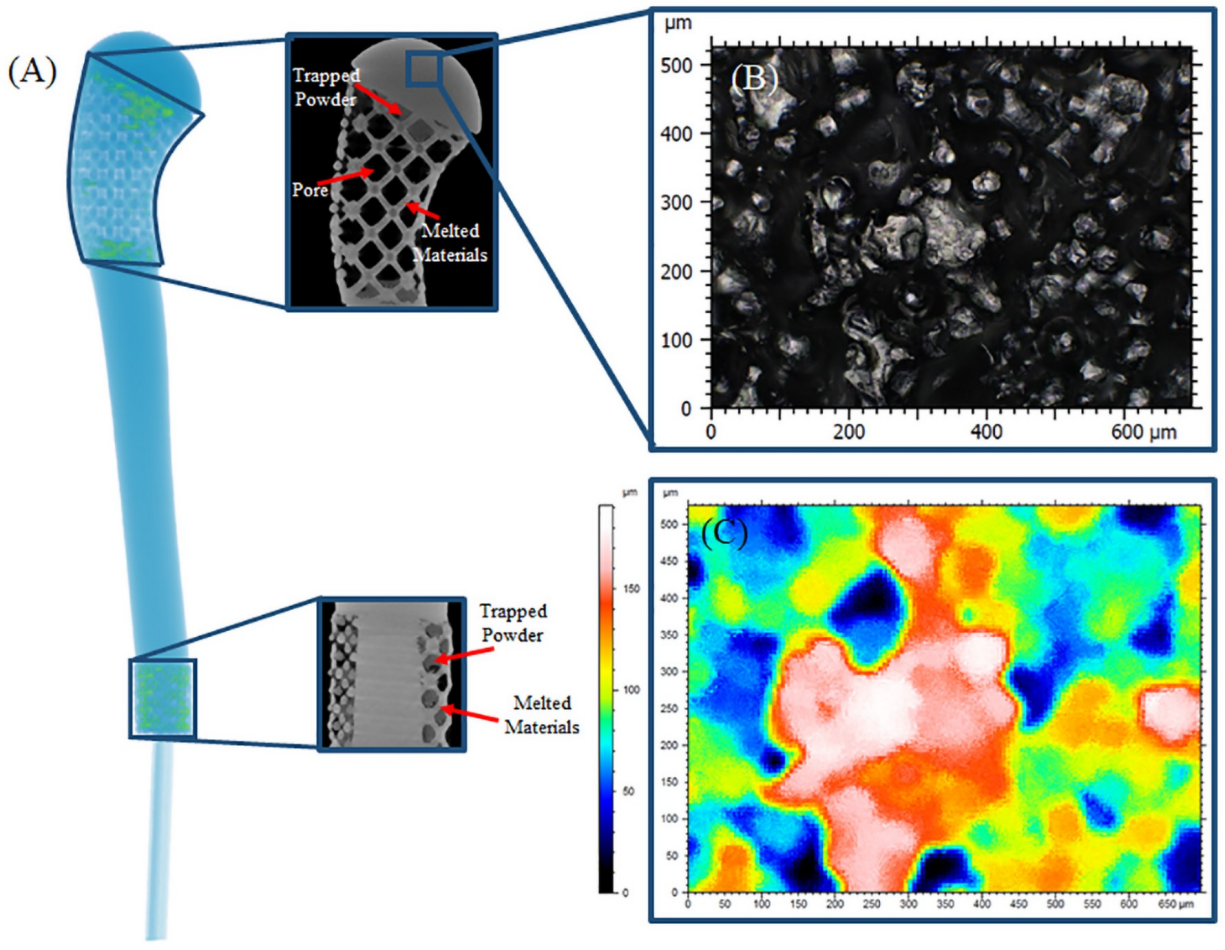
(A) Presence and distribution of trapped powder in porous regions of the prosthesis. In the CT images, trapped powders had halftone grayscale, which is between the melted powder and the void space (i.e., pore) grayscale values. Representative (B) all-in focus image showing the surface texture of the as-built EBM patient-specific prosthesis, and (C) associated height map.

The patient-specific prosthesis was scanned using a micro computed tomography (micro-CT) scanner (v|tome|x L300 multi-scale microCT system, General Electric, Boston, MA, USA) before and after biomechanical testing. In this study, 300 kV microfocus X-ray was employed to scan the prosthesis before and after cyclic loading with a voxel size of 100 *μm*. Images from the micro-CT revealed the presence of trapped powders in some of the pores. In order to automate the segmentation of voxels with varying grayscale (i.e. solid, trapped powder, and pores) in Avizo (V9.0, FEI Houston, Hillsboro, OR, USA), the Trainable Weka Segmentation (TWS) [34] plugin in ImageJ [35] was employed. The TWS tool uses an open source machine learning software Weka (University of Waikato, Hamilton, NZ) to train a Random Forest classifier based on user-defined representative image stack [36, 37]. Segmented images were reconstructed in 3D for further analysis in Avizo (Fig 4A). Presence of the trapped powder in the designed porous regions was quantified based on percent volumetric fraction (Fig 4A).

Morphological characterization was performed on the segmented data using the pore-network tool in Avizo. Porosity at both the surgical neck and the distal interface was evaluated before and after biomechanical testing. Pore size was computed from the diameter of the maximum sphere that fits within the pores. Pore connectivity and strut size were analyzed using thickness map function in Avizo. It should be noted that strut size measurements from micro-CT data were performed by excluding the trapped powders in order to isolate the effects of load-carrying solid struts. However, pore size, porosity, increase in surface area (ratio of the designed porous part surface area to the substituting solid object (A_P_/A_S_)), and tortuosity calculation included the presence of trapped powders to evaluate the true morphological parameters of the as-built AM prosthesis.

#### 2.5.2. Surface morphology

Surface morphology of the 3D printed prosthesis was evaluated using an optical microscope (i.e., non-contact surface inspection) (Axio Imager M2m, Carl Zeiss, Germany) (Fig 4B and 4C). Due to difficulties in performing optical topography on curved surfaces, a cubic specimen fabricated on the same build with the prosthesis was used for surface roughness analysis. In this study, confocal z-stacks were converted into height maps (Fig 4C) which were used to determine ISO (international organization for standardization) conforming surface roughness [38]. Surface roughness was computed from height maps across cut-off length of 4.5 mm. A low-pass Gaussian filter with 0.8 mm cut-off frequency was applied to the height stacks to determine the surface roughness. In other words, in order to obtain the roughness profile, the low passed filtered height profile was subtracted from the primary height profile. Height and amplitude parameters including: maximum peak height (*S*_*p*_, *R*_*p*_), maximum valley depth (*S*_*v*_, *R*_*v*_), maximum surface height (*S*_*z*_, *R*_*z*_), arithmetic mean surface height (*S*_*a*_, *R*_*a*_), and root mean square surface height (*S*_*q*_, *R*_*q*_) were reported in accordance with ISO-25178 and ISO-4287.

## 3. Results

### 3.1. Biomechanical testing results

**Fig 5:**
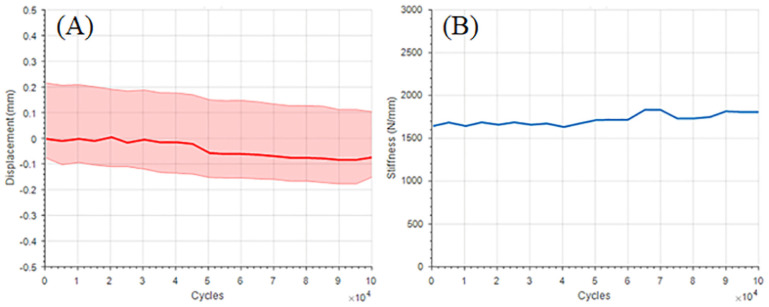
Cyclic loading results: (A) Displacement (of overall construct) vs number of cycles (shaded region represent variation in max and min of displacement at every cycle; and the solid red line is the mean displacement for every cycle), and (B) cyclic stiffness vs. number of cycles.

The prosthesis did not experience catastrophic failure during the cyclic loading. Results from the biomechanical test indicated that the patient-specific AM prosthesis experienced 82 *μm* permanent deformation of the overall construct (as measured net crosshead displacement throughout the course of testing) through 10^5^ applied cycles (Fig 5A). Slight increase in the stiffness (approximately 150 N/mm) was observed over the applied number of cycles. Change in the cyclic stiffness of the prosthesis is shown in Fig 5B.

### 3.2. FEA results

**Fig 6:**
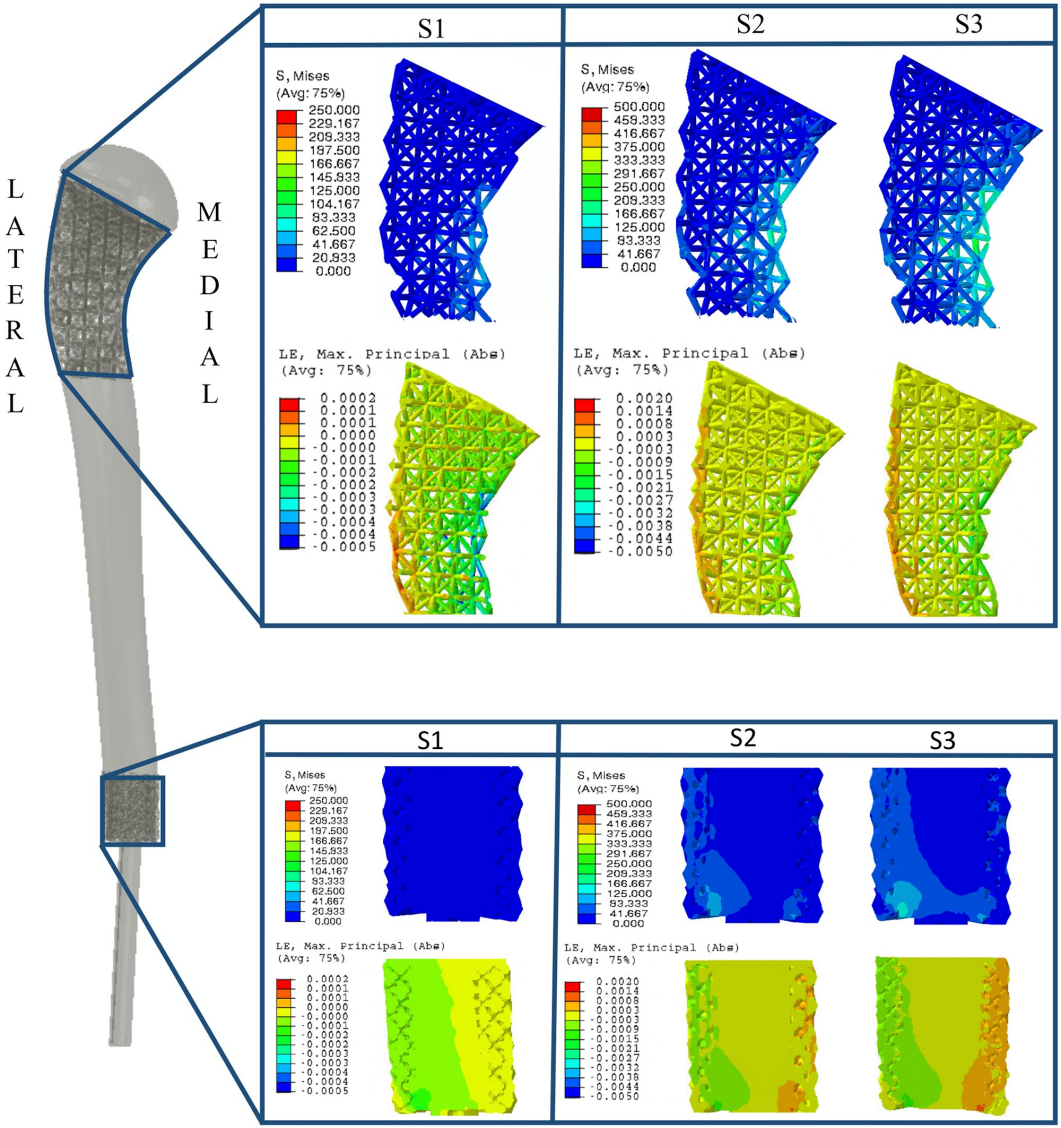
FEA results: Von Mises stress (MPa) and maximum principal strain (absolute values) distributions across porous regions of the patient-specific AM prosthesis.

FE model associated with the loading condition S1 was validated with experimental cyclic loading. Predicted stiffness (based on S1 model = 2114 N/mm) was 14.5% higher than the experimental result (1846 N/mm) which corresponded to the mean stiffness of initial 5–10 cycles (first four applied cycles were considered as pre-conditioning [23, 39, 40]). Additionally, stress and strain distribution at the porous regions of the AM prosthesis were evaluated (Fig 6). Across all loading conditions, the medial aspect of the porous surgical neck had the highest stress concentration followed by lateral aspect of the distal interface. However, the maximum von Mises stress was well below the yield stress of Ti-6Al-4V (1100 MPa) and the estimated fatigue strength across all conditions (Fig 6). In loading condition S1, the strain in z-direction (Fig 3) showed tensile and compressive displacements at the lateral and medial aspect respectively (Fig 6). In the S2 and S3 conditions, medial compressive displacement was mainly observed with little or no tensile displacement at the lateral side (Fig 6).

### 3.3. Characterization

#### 3.3.1 Morphology

AM fabrication and post-cleaning process for the designed prosthesis took less than 24 hours. Micro-CT scans of the prosthesis (after sand blasting) revealed presence of residual trapped powders in the porous regions. These trapped powders had 19.44% of the designed void space at the surgical neck and distal interface of the implant together (Fig 4A). Morphological parameters showed deviations between as-built and as-designed implant as noted in Table 1. The as-built pore size, which was measured using pore-network analysis on the micro-CT data, was found to be greater than the as-designed values. Moreover, morphological analysis on the micro-CT data showed that as-built porosity was 70.94% and 51.26% when compared to as-designed porosities of 76.8% and 69.8% at the surgical neck and the distal interface, respectively.

**Table 1:** Morphological parameters at the porous regions of the prosthesis.

	Region	Pore size (mm)		Strut size (mm)		Increase in Surface Area (A_P_/A_S_)		Tortuosity
		As-designed	As-built	As-designed	As-built	As-designed	As-built	As-built
Before Testing	Neck	1.2–3.4	5.48 ± 1.52	0.8–2.0	2.07 ± 1.20	3.81	3.45	1.39
	Distal	1.4	2.73 ± 0.91	1.0	1.49 ± 0.87	2.30	1.99	1.18
After Testing	Neck	—	5.17 ± 1.52	—	2.10 ± 1.16	—	3.45	1.29
	Distal	—	2.61 ± 0.54	—	1.52 ± 1.02	—	1.95	1.15

Parameters in range of values were reported in mean ***±*** standard deviation. “—” was used when additional analysis was not applicable.

#### 3.3.2. Surface roughness

Surface morphology analysis (i.e., height and amplitude roughness parameters) was performed on the patient-specific prosthesis using optical non-contact microscopy (Table 2). As-built surface roughness parameters, *S*_*a*_ *and R*_*a*_, in this study were within the recommended range of surface roughness for metallic load-bearing implants [41–44] to promote osseointegration and tissue-implant fixation.

**Table 2:** Measured roughness parameters, height surface roughness (ISO 25178) as well as amplitude-roughness (ISO 4287) after Gaussian filter with a cut-off value of 0.8 mm was applied.

Parameter	*S*_*p*_, *R*_*p*_	*S*_*v*_, *R*_*v*_	*S*_*z*_, *R*_*z*_	*S*_*a*_, *R*_*a*_	*S*_*q*_, *R*_*q*_
Value (μm)	88.1, 63.8	103, 80.4	191, 144.0	33.3, 29.0	40.0, 35.0

S_*p*_ (R_*p*_): maximum peak height; S_*v*_ (R_*v*_): maximum valley depth; S_*z*_ (R_*z*_): maximum peak to valley height; S_*a*_ (R_*a*_) arithmetical mean height of the surface; S_*q*_ (R_*q*_): root mean square height of the surface.

## 4. Discussion

### 4.1. Biomechanical performance

In this study, validated FEA and experimental analysis showed that AM prosthesis, based on the proposed design workflow, can withstand physiologically-relevant loads without experiencing apparent catastrophic failure. In this study, commonly used BCC open-cellular structure [17] was employed in the design process of the patient-specific prosthesis. Such topology (i.e., BCC) exhibit sharp self-intersecting strut junctions which are areas of stress concentrations. However, these sites of stress concentration did not cause catastrophic failure of the designed patient-specific implant since the magnitude of the applied stress was not sufficient. However, in order to achieve smoother stress distribution and reduce the risk of fatigue failure, sharp strut junctions could be replaced with hyperbolic (or smooth continuous) curvature. Minimal surfaces [45] exhibit such a property and could be incorporated into the design of patient-specific porous AM prosthesis instead of self-intersecting mechanical structures. In addition, increasing the strut thickness (or wall-thickness) while staying within the recommended pore size range could further reduce the fatigue failure. Previous studies [46–49] have shown that bone ingrowth is dependent on the sign of surface curvature and minimal surfaces which mimic native bone tissue morphology can enhance biological responses such as osteoblast cell adhesion, growth and differentiation.

The cyclic biomechanical testing of the AM prosthesis resulted in a permanent compressive displacement of 82 *μm* which is substantially lower than the accepted upper threshold of micromotion without disturbing the natural bone tissue healing process. From the FE analysis (in the S1 loading condition) highest stresses were located at the medial aspect of the porous surgical neck. This observation is attributed to the bending stress since the medial aspect of the surgical neck also experienced higher strain. In addition, slight increase in the cyclic stiffness was observed which could be attributed to small angulation of the prosthesis and/or compliances in the fixtures during the experiment.

In this study, linear elastic isotropic material model was used for the AM Ti-6Al-4V patient-specific prosthesis which may not accurately replicate the material model for AM implants (although anisotropy is significantly less prominent in EBM process). Additionally, effects of the muscle forces to the biomechanical behavior of the prosthesis were ignored. In order to gain better insights into the biomechanical responses of patient-specific implants, muscle reaction forces need to be integrated into the FEMs. This study was also limited to one sample size, in order to gain statistical power, larger sample size needs to be used.

Similar to other investigations [50–52] into the effects of as-built surface of metal AM implants on the osseointegation characteristics, this study was limited to in-vitro and in-silica biomechanical and morphological analysis. Commonly accepted theory is that the greater surface roughness leads to migrations of the cells into the pores, and proper strain levels at the tissue-implant interface results in bone formation [41]. However, effects of as-built AM surface roughness on the tissue-implant interaction mechanism are still unclear. In order to gain deeper understanding on interaction mechanism between AM implant and tissue, future studies are required to explore the quantitative correlation between the implant’s surface roughness, effective surface area, bone ingrowth, and strains at the tissue-implant interface.

### 4.2. Physical and morphological properties

This study provides preliminary evidence supporting patient-specific AM prostheses for the reconstruction of large bone defects in oncology patients, specifically pediatric patients. The proposed patient-specific prosthesis included volumetric porous structures at the surgical neck and distal interface. Large pore size at the surgical neck could potentially facilitate suturing of the tendons into the implant while also allowing for direct tissue ingrowth onto the implant. Similarly, the distal porous interface allows for bone ingrowth since the distal dissection usually carried out to the level of osteotomy to prevent osteonecrosis of the remaining bone segment. Another unique design feature in the studied implant was the somewhat triangular-shape of the stem which would lock the rotational alignment of the implant due to the matching shape with the triangular marrow cavity. Together, porous distal interface and the marrow cavity-shape stem could reduce the risk of future periprosthetic loosening. Although direct clinical application was beyond the scope of this study, the AM patient-specific prosthesis was designed to fit the anatomy without the need for glenoid replacement given the opportunity of suturing the host rotator cuff and capsule to the porous surgical neck. And if the glenoid were to become arthritic in the future, conversion to a total shoulder arthroplasty with glenoid resurfacing could be carried out. It is worth highlighting that the proposed AM prosthesis may only be considered in patients near the end of growth or skeletal maturity in order to avoid any limb length discrepancy issues associated with growth.

In this work, EBM process was employed to manufacture the designed patient-specific prosthesis in less than 24 hours. The EBM process was performed at an elevated temperature of 750°C (layer temperature) which results in stress relieved parts (cool-down) with material, mechanical, and chemical properties potentially better than cast and wrought forging [53]. Therefore, in contrast to laser powder bed fusion parts, EBM parts do not require post- heat treatment for residual stress relief purposes.

Structural and morphological comparisons between as-designed and as-built (using micro-CT) revealed the presence of trapped powders in porous regions which contributed to the observed deviations in the morphological parameters such as pore size, porosity, tortuosity, and effective surface area. Concentration of the trapped powders was small in the surgical neck, when compared to the distal interface porous region, and was only found in pores that were close to solid surfaces (i.e., solid-porous interfaces). However, due to small pore size in the distal interface compared to the surgical neck, increase in volume of the trapped powders was observed. Reduction in porosity and effective surface area from as-designed to as-built is partly attributable to the presence of trapped powders. When comparing micro-CT data between pre- and post-experiment, negligible variations (<6%) in morphological parameters were found. In addition, no structural deformities or micro-damage was observed in the micro-CT data at 100 *μm* voxel resolution. Large deviation in the calculated morphological parameters (between as-designed and as-built) could be partly attributed to the effects of sandblasting. During the sandblasting process, exterior struts are more prone to undesired material removal when compared to interior cellular structures. This may have caused variation in strut size, interior struts having larger strut diameter with exterior struts showing smaller diameter. Such variations resulted in large pore size deviations across each porous region. This partly explains the higher average pore size values (with relatively large standard deviation) in the 3D reconstructed as-built model as compared to the as-designed values. In addition, ineffectiveness of the sandblasting process in completely removing trapped powders could be associated to line-of-sight limitations that restricts the permeability of sand particles within the pore spaces. Micro-CT analysis of the pre- and post- biomechanical testing of the prosthesis showed minor deviation in the morphological parameters considering that post-process the micro-CT data is a subjective process. Despite that, it should be acknowledged that presence of trapped powder particles in the implant needs to be addressed. Further investigation in establishing an effective post-processing protocol is recommended to eliminate any entrapped powder particles in the implant after vigorous cleaning and sterilization process. Additionally, future studies should further investigate alternative approaches to minimize or eliminate presence of trapped powder particles in such implants, either by replacing beam-based unit cells with surface-based topologies (e.g., minimal surfaces) [54, 55] or changing the manufacturing method.

## 5. Conclusion

This study provides a systematic workflow for the design-fabrication-evaluation of patient-specific prosthesis for reconstruction of the proximal humerus following tumor resection, specifically in pediatric patients. Following conclusions are drawn from the outcomes of this study:

- Both validated FEA and experimental analysis showed that the proposed patient-specific AM prosthesis provides adequate biomechanical strength under physiologically relevant cyclic loading conditions without experiencing catastrophic or structural failure.
- Micro-CT evaluation of the AM implants provides comprehensive information on morphological parameters including deviations in morphological parameters between as-designed and as-built implants. Those deviations could be partly attributed to trapped powder inside the porous regions of the prosthesis.
- Self-intersecting lattice structures (such as BCC) are prone to high stress concentrations at the strut junctions under cyclic loading.
- Additional clinical studies are required to evaluate the effectiveness of patient-specific AM prostheses to eliminate residual deformities and improve implant fixation. This could be valuable in uniquely challenging cases such as pediatric bone tumor resection.
